# Inconclusive Syphilis Results: A Retrospective Review of Public Health Results

**DOI:** 10.1155/2020/5642952

**Published:** 2020-08-14

**Authors:** Patrick O'Byrne, Leslie Tilley, Dara Spatz Friedman, Lauren Orser

**Affiliations:** ^1^University of Ottawa, School of Nursing, 451 Smyth Road, Ottawa, Ontario K1H 8M5, Canada; ^2^Ottawa Public Health, Infectious Diseases and Sexual Health Services, Ottawa, Canada; ^3^Ottawa Public Health, Ottawa, Canada; ^4^University of Ottawa, School of Epidemiology, Ottawa, Canada

## Abstract

In the context of increasing syphilis incidence in many Western countries, we sought to better understand the frequency and outcomes associated with inconclusive serologic syphilis results. To accomplish this, we reviewed all inconclusive results that arose from an indeterminant confirmatory treponemal screen (specifically the *Treponema pallidum* particulate agglutination test), which were reported to Ottawa Public Health from January 1, 2019, through December 31, 2019. Our case review identified that 52 persons generated such test results during the study period, of whom 44.4% were cases requiring treatment, 46.3% were persons without new risk factors or symptoms of syphilis who had been previously treated for this infection, and 9.3% were not syphilis. Overall, these untreated syphilis cases accounted for 8.6% of all new syphilis diagnoses in our local jurisdiction during the study period. These results highlight that case investigation and prompt management of inconclusive syphilis results is an appropriate public health and clinical approach and that such a strategy could contribute to efforts to reduce increasing syphilis incidence.

## 1. Background

Syphilis, caused by the spirochete *Treponema pallidum*, is a sexually transmitted bacterial infection of public health concern and, in Canada, is considered infectious if present for less than 12 months and noninfectious if present for longer [1, 2]. This infectious period comprises the stages of primary, secondary, and early latent syphilis, which include mucocutaneous lesions (e.g., chancre, rash, and condylomata lata) and flu-like symptoms [1, 2]. The noninfectious period comprises late latent syphilis, which is asymptomatic, and tertiary syphilis, which often affects the central nervous system, heart, and/or skin, although it can affect any organ system [1, 2].

While the rates of infectious syphilis had become so low by the year 2000 that the US Centers for Disease Control and Prevention speculated that this infection could be virtually eradicated by 2005 [3], syphilis reemerged over the last 10 years [4]. Since 2010, the rates of syphilis have increased by an average of 151% in Canada, mostly in men who have sex with men and, to a lesser extent, in heterosexual females, with corresponding increases in congenital syphilis [4, 5]. This situation has required that clinicians and public health workers learn how to prevent, diagnose, and manage syphilis—which was something many practitioners had never done before.

Complicating syphilis management is that its diagnosis is usually based on the interpretation of various serologic antibody tests, with none being a direct test for syphilis [1]. While direct specimen collection from identifiable syphilitic lesions (e.g., chancres or condylomata lata) is one way to diagnose syphilis, this testing modality is often not possible because persons are asymptomatic, lesions cannot be visualized because they are internal (e.g., in a person's rectum or on the cervix), symptoms are not recognized by patients or providers, or the testing is unavailable in a specific jurisdiction. As such, in most cases, clinicians usually must interpret a panel of three antibody results, two of which are treponemal-specific (although unable to distinguish between venereal and endemic treponemes) and one is nontreponemal and rises in response to tissue damage that is nonspecific for syphilis [1].

In Canada, such testing follows what is known as the reverse algorithm and involves a qualitative treponemal screen (often an enzyme immunoassay (EIA)), which, if positive based on manufacturer-established signal to cutoff ratios, is followed reflexively by two other tests: (1) a confirmatory qualitative treponemal test that reacts to a different protein than the screening EIA (often a *Treponema pallidum* particle agglutination (TPPA) test, which can be reactive or nonreactive, or indeterminate when equivocal) and (2) a quantitative nontreponemal test (e.g., rapid plasma reagin (RPR), which is a dilution-based test that produces titres) [6]. In combination, these tests can indicate infection with sexually transmitted syphilis, but do so without differentiating between a new untreated infection, an older treated infection, and an older untreated infection. Such distinction is based on history and examination [2]. Nonetheless, the main benefits of the reverse testing algorithm are that the screening EIA is highly sensitive for syphilis (even in early infection) and that reflex testing often helps rule out false positives.

Despite these benefits, one shortcoming of the reverse testing algorithm is that it can yield inconclusive results [1, 2, 6], which arise from one of the two combinations of test results: (1) a reactive EIA screen, a nonreactive RPR, and an indeterminate TPPA, or (2) a reactive EIA screen, a reactive RPR, and a nonreactive TPPA [7]. Herein lies the issue: the differential diagnosis for an inconclusive syphilis result from the reverse algorithm can be complicated, especially for practitioners who are less familiar with syphilis. Indeed, the interpretation of such an inconclusive syphilis result has five possibilities. The person with this result may have (1) a new untreated infection (with low antibody levels due to active seroconversion), (2) an older treated infection (with waning antibodies posttreatment, which is more common when syphilis is treated earlier after acquisition), (3) an older untreated infection (with naturally waning antibodies), or (4) an endemic treponemal infection (which is indistinguishable from syphilis) [1]. The inconclusive results may also (5) be a false-positive result due to immunologic or inflammatory conditions (see Table 1).

While the Public Health Agency of Canada (PHAC) [2] has a comprehensive table to help clinicians interpret syphilis results, this resource is limited to the list of differential diagnoses listed in Table 1 for inconclusive syphilis results, without indication of the frequency of causes or rates of occurrence among different populations (e.g., men who have sex with men, new immigrants). In addition, PHAC recommends repeat syphilis testing 2–4 weeks after the initial test to monitor for changes in results, but makes this recommendation without providing guidance about how to clinically manage results that do not change or about what counselling or follow-up should occur while awaiting the results of this repeat testing [2]. Similarly, the PHAC guidelines do not give recommendations for public health staff regarding case investigation, such that public health staff are left to interpret inconclusive laboratory results with little empirical guidance. This is particularly concerning in the context of Canada-wide increases in the incidence of syphilis [4, 5].

To address these knowledge gaps, we undertook a retrospective review of all inconclusive syphilis results that arose in Ottawa from indeterminant TPPAs. Our objectives were to understand the outcomes of these results at follow-up (i.e., how many of such results were false positives versus new cases versus older treated or untreated cases?), to determine if different outcomes were more common among specific populations (e.g., do inconclusive results mean the same thing in different patient populations, for example, men who have sex with men versus persons who immigrated to Canada?), and to provide clinical guidance for these results (how might clinicians and public health staff manage these results in the future?).

## 2. Methods

In Ontario, all syphilis testing occurs at the Public Health Ontario Laboratory (PHOL), with any reactive syphilis screen (EIA) result being reportable to the local public health unit. Because inconclusive syphilis results are always accompanied by a positive EIA, they, too, are reportable, irrespective of RPR and TPPA result [8]. To better understand the characteristics of persons with inconclusive results arising from a reactive EIA screen, a negative RPR, and an indeterminate TPPA and the outcomes associated with these results, we undertook a retrospective review of inconclusive syphilis results that were reported to Ottawa Public Health from January 1, 2019, through December 31, 2019. This look-back started with all syphilis results during the study period with an inconclusive TPPA being extracted from the Ontario Ministry of Health and Long Term Care integrated Public Health Information System (iPHIS)—which is the provincial reportable infectious disease database in Ontario. We then manually reviewed each case associated with these results, including subsequently reported test results and associated clinical stage of syphilis (i.e., infectious versus noninfectious). As recommended by PHAC, we sought to identify changes in syphilis results (e.g., changes in TPPA result) to determine the evolution of results and the indication of a potential infection [2]. As part of this process, we extracted information about why testing was ordered and if symptoms were present at the time of testing. We also extracted risk factor information, as collected by the public health unit.

In Ontario, classification of syphilis cases follows Public Health Ontario case definitions [9], which match those from PHAC [10]. Based on these definitions [9, 10], infectious cases include primary, secondary, and early latent syphilis, and noninfectious syphilis includes late latent syphilis, latent syphilis of unknown duration, and tertiary syphilis.

## 3. Results

In Ottawa, from January 1, 2019, through December 31, 2019, 279 persons were diagnosed with syphilis, of whom 171 had infectious syphilis (primary, secondary, or early latent), 56 had noninfectious syphilis (all late latent, none with tertiary syphilis), and 6 were pending classification. Another 46 persons were diagnosed with latent syphilis of unknown duration, meaning that clinicians could not determine if patients had acquired syphilis within more or less than the last 12 months. Among individuals diagnosed with syphilis (any stage), 54 persons—for a rate of 5.1/100,000—produced inconclusive syphilis results during the 12-month study period.

Of the 54 persons with inconclusive test results, 24 required treatment (*n* = 13 as infectious cases and *n* = 11 as noninfectious cases). The infectious cases were staged based on follow-up results becoming fully positive (i.e., reactive TPPA with or without a rise in RPR) or the presence of unequivocal syphilis lesions at the time the person was first tested for syphilis. Notably, two persons with inconclusive results were diagnosed with and treated for syphilis based on symptoms 2–4 weeks prior to their first inconclusive test results; these two persons had negative test results at that first visit. Both of these cases also denied new potential exposures to syphilis following treatment, meaning that their subsequent inconclusive results likely represented stunted seroconversions after treatment at their first clinical visit. Those who were staged with late latent infections (*n* = 11) all had historical risk factors (nonrecent exposures) for syphilis and denied previous treatment. As well, everyone determined to be an infectious case was a male who reported same-sex partners, whereas, for the noninfectious cases, of those with identified risk factors, one-third were men who have sex with men and two-thirds had immigrated to Canada. As such, 44.4% (*n* = 24/54) of persons with a first-time inconclusive syphilis result required treatment, with 24.1% (*n* = 13/54) of these persons being new cases of infectious syphilis (see Table 2). Moreover, the 24 persons with inconclusive syphilis results who required treatment constituted 8.6% (*n* = 24/279) of all syphilis diagnoses in Ottawa during our 12-month study period.

Furthermore, of these 54 persons with inconclusive test results, 46.3% (*n* = 25/54) had no recent risk factors for syphilis and had documented previous treatments or reported such prior treatment when informed about their inconclusive results. Additionally, two were diagnosed with an endemic treponemal infection by infectious disease specialists and three were determined to have false-positive results based on seroreversion on subsequent results or a lack of potential exposures.

## 4. Discussion

We undertook a retrospective review of inconclusive syphilis results in Ottawa from January 1, 2019, to December 31, 2019. From this, we identified 54 inconclusive syphilis results, of which 46.3% (*n* = 25) were in persons with previously treated infections, 44.4% (*n* = 24) were in persons who required treatment for infectious (*n* = 13/24) or noninfectious (*n* = 11/24) syphilis, and 9.3% were in persons with false-positive results. Notably, these 24 new cases accounted for about one in 12 syphilis cases diagnosed in Ottawa during the study period. These results raise three noteworthy points for discussion.

First, these findings add to what is known about inconclusive syphilis results. While extant PHAC guidelines [2] list the outcomes we identified (i.e., older previously treated infection, older untreated infection, new infection, endemic treponemal infection, and false positive), our data add important clarifications about these causes and the affected population groups. To the best of our knowledge, there have only been previous case reports [11] and clinical expert guidance [2] for these outcomes, not retrospective reviews of results. Other studies also reviewed false-positive results related to the RPR [12, 13], not the reverse algorithm. Our findings, in contrast, constitute what we believe is the first study to categorize inconclusive syphilis results for an entire public health unit based on the frequency of the various possible causes of such results and the risk factors associated with these outcomes.

Second, that there were 54 persons (5.1/100,000) with inconclusive results in Ottawa during our 12-month study period highlights that these results occurred frequently enough that clinicians and public health staff should be familiar with what they mean and how to manage them. Our results also showed that the minority of inconclusive results were false positives. Instead, most of the inconclusive results we reviewed occurred in persons previously treated for syphilis, followed closely by persons with new or older untreated infections. New infections were almost exclusively in men who have sex with men, whereas older infections had a larger proportion in those who had immigrated to Canada. That the new infections occurred in men who had sex with men, however, was possibly an outcome of the fact that most incident syphilis cases in Ottawa were in this group during the study period. That is, we likely found more inconclusive results among men who have sex with men because, on the one hand, the burden of infection was higher (and thus more infections were being identified), while, on the other hand, more frequent testing also occurred among this group (and thus earlier identification of infection was also more likely). This finding might change, were the groups most affected by syphilis to be different; for example, if the incidence rate of infectious syphilis were higher among females with opposite-sex sexual partners. However, more research is required to confirm this point.

Third, our results raise important points for clinical and public health practice. On the public health side, it seems that public health units should investigate inconclusive syphilis results to determine if they constitute new cases. This does not mean that an inconclusive test result should automatically be considered indicative of infection; however, it also does not outright preclude it from being one. In other words, an inconclusive result is neither a case nor not a case; it is a result that warrants investigation to determine categorization. According to Public Health Ontario [9] and PHAC [10] case definitions, an inconclusive syphilis result in a person without a history of syphilis who has unequivocal primary syphilis lesions meets case definition, as this inconclusive syphilis result would have arisen from a reactive treponemal result (i.e., EIA screen), albeit indeterminate confirmatory testing, in someone with symptoms. In contrast, such a result in an asymptomatic person who was previously treated for syphilis with no recent risk factors and a history of previous inconclusive test results might not be a case. What is required is an investigation: Was the person symptomatic? Was there a history of previous syphilis and treatment? Does the person have new risk factors for syphilis? The answers to these questions should guide practice.

Our results reinforce the importance of this public health investigation: from our 54 persons with inconclusive results, 44.4% (*n* = 24) required treatment, with 54.2% (*n* = 13/24) of those requiring treatment being new infectious cases. Investigating inconclusive syphilis results could improve case finding, help identify sexual partners who require treatment, and disrupt onward transmission [14]. These outcomes could likely also occur quickly after infection, as the inconclusive results in our sample that were infectious cases likely arose from partial seroconversions during early infection. Based on our experience, potential public health benefits could also occur without many additional resources, as nearly half (46.3%, *n* = 25/54) of the persons with inconclusive results in our jurisdiction either recalled prior treatment when asked or had this treatment documented in the public health record (i.e., in iPHIS). Public health investigation of such results therefore seems prudent and feasible.

On the clinical side, these results highlight that clinicians should interpret inconclusive syphilis results considering patient history and examination and epidemiologic risk. As is the case for public health unit investigation, clinicians should ask, does the patient have symptoms? Does s/he/they belong to a group affected by syphilis? Are there previous test results for syphilis, and what are these results? Our results highlight that a new inconclusive result in a gay man with prior negative syphilis results likely represents a new case, whereas a new and repeated inconclusive result in a person who emigrated from an area where syphilis is endemic may have a late latent infection. On this point, clinicians must remember two important items: (1) repeat inconclusive results do not rule out late latent syphilis infections, and (2) the assumption that inconclusive syphilis results are false-positive results could leave patients at risk for tertiary syphilis because s/he/they were not treated when they should have been. It might therefore be prudent to consider treatment in these cases to eliminate the risk of future sequelae from a potentially untreated infection. Our results suggest that this approach is reasonable: in the context of increasing rates of syphilis across Canada, while we identified that half of our inconclusive results were in people with previously treated infections, almost all of the remaining cases of inconclusive results involved persons with new or older infections that warranted treatment. In other words, while PHAC [10] guidelines appropriately indicate a potential need for repeat syphilis testing 2–4 weeks after a first inconclusive test result, our results suggest that clinicians should not presume that unchanging inconclusive results always constitute false-positive results. Instead, despite PHAC guidelines stating that “if repeat serology is unchanged this is likely to be a false positive,” any possible history of potential exposure to syphilis, no matter how remote, should be considered to rule out untreated late latent infections. A review of treatment at the point of testing should also occur. Identification of previous treatment without recent risk factors could easily identify a remotely treated infection, whereas prompt treatment for someone with risk factors for acquiring syphilis who never previously received treatment could quickly eliminate infectious syphilis and contribute to population level reductions in incidence. That is, our results highlight that it is reasonable to promptly treat a person with risk factors for syphilis at the point of a newly obtained inconclusive result when this person had a previously documented negative syphilis test result. Additionally, all of this might reduce needless referrals to infectious disease specialists.

Our results, however, must be interpreted in light of certain limitations. First, our study was restricted to one urban Canadian centre with a population of 1 million. These results may be contextually bound by syphilis epidemiology in Ottawa, where (1) there has been a 4-fold increase in the rates of infectious syphilis between 2014 and 2019 and (2) most new syphilis infections are in men who have sex with men. If such results would apply in other settings and scenarios is unknown. Second, while we reviewed inconclusive results for 54 persons, our 12-month study period was short and our study sample was too small to perform statistical testing. Future studies need to better examine the differences between persons with different inconclusive test results. As well, future studies need to engage in more in-depth clinical reviews, moving beyond our reliance on public health system data. This information could inform public health and clinical efforts. Lastly, our analysis of inconclusive results related exclusively to those generated from indeterminate confirmatory test results (i.e., TPPA). Future analyses need to determine if the same results arise for inconclusive results from a reactive screening EIA and RPR with a negative TPPA.

Notwithstanding these limitations, our data constitute what we believe is the first study to classify all inconclusive syphilis results reported to a public health unit. This adds to the available literature on this topic, which primarily arises from case reports. Our results, in turn, suggest that public health units should investigate inconclusive results to determine if these results meet case definition and that clinicians need to interpret these results for each patient, erring on the side of providing treatment to persons with risk factors to thwart onward transmission and the potential future development of tertiary syphilis. Considering markedly increased rates of syphilis, such efforts might be useful practice guidance and could contribute to the rates of syphilis again decreasing. In the meantime, routine syphilis testing is advisable, with expert consultation as needed.

## Figures and Tables

**Table 1:** Inconclusive results and interpretations.

Test		Interpretations
Treponemal			Nontreponemal
EIA (screen)	TPPA (confirmatory)		RPR (confirmatory)
Reactive	Nonreactive	Indeterminate	(i) New infection (ii) Older untreated infection (iii) Older treated infection (iv) Other nonvenereal treponemal infection (v) False positive
Reactive	Reactive (with titre)	Nonreactive	

**Table 2:** Summary of results.

Category	Subcategory	Number	Percentage
Cases
	Infectious	13	24.1
	Noninfectious	11	20.4
	Row total	24	44.4

Previously treated		25	46.3

			
Not syphilis
	Endemic treponemes	2	3.7
	False positive	3	5.6
	Row total	5	9.3
Total		54	100

## Data Availability

The data in this study arose from the Government of Ontario, Ministry of Health and Long Term Care's Integrated Public Health Information System.
